# Vitamin D as a Modifiable Risk Factor in Schizophrenia a Systematic Review

**DOI:** 10.3390/biom15081094

**Published:** 2025-07-28

**Authors:** Jadwiga Mosiołek, Bartosz Mosiołek, Agata Szulc

**Affiliations:** 1 Faculty of Health Sciences, Medical University of Warsaw, Żwirki i Wigury 61, 02-091 Warsaw, Poland; 2 Mazowieckie Specjalistyczne Centrum Zdrowia im. Prof. Jana Mazurkiewicza, Partyzantów 2/4, 05-802 Pruszków, Poland; 3 Faculty of Medicine, Medical University of Warsaw, Żwirki I Wigury 61, 02-091 Warsaw, Poland

**Keywords:** schizophrenia, vitamin D, deficiency, psychosis, cholecalciferol, calcitriol

## Abstract

The etiology of schizophrenia remains poorly understood. Although certain risk factors have been identified, effective preventive measures are still lacking. This study investigates potential preventive methods while focusing on the role of vitamin D and its status. The role of malnutrition in schizophrenia risk was first identified in studies on the Dutch Hunger Winter. Vitamin D deficiency was hypothesized as a contributing factor shortly thereafter. This review aims to explore the correlations between vitamin D deficiency at various life stages (maternal, neonatal, adult) and schizophrenia risk, as well as its effects on pharmacokinetics, neurobiology, bone health, and metabolic syndrome. The studies were retrieved from two indexed databases, PubMed and Web of Science, following PRISMA guidelines and included studies published between 2000 and 2024. No correlation was found between maternal vitamin D levels and schizophrenia in offspring while a positive correlation was observed between low neonatal vitamin D levels and schizophrenia in later life. Approximately half of the studies on adults reported mean vitamin D concentrations of below 20 ng/mL which were negatively correlated with gray matter volume and bone health while positively correlated with the prevalence of metabolic syndrome. Additionally, vitamin D levels were also found to correlate with antipsychotic drug concentrations.

## 1. Introduction

The discovery of vitamin D dates back to the early 1600s with initial descriptions of human deficiency diseases: rickets in children and osteomalacia in adults. Approximately 300 years later, between 1900 and 1920, physicians and biochemists identified the chemical structures of the two main forms of vitamin D, namely vitamin D2 and vitamin D3 [1]. The term “Vitamin D” was first introduced in 1922 by McCollum et al., who suggested the existence of a factor promoting calcium deposition [2]. The year 1967 marked the discovery of the active form of vitamin D, 1,25-dihydroxyvitamin D (1,25-(OH)2D). Since then, ongoing research has continued to uncover new insights into cellular mechanisms, functions, and vitamin D-related human diseases [1].

### 1.1. Vitamin D Metabolism

**Figure 1:**
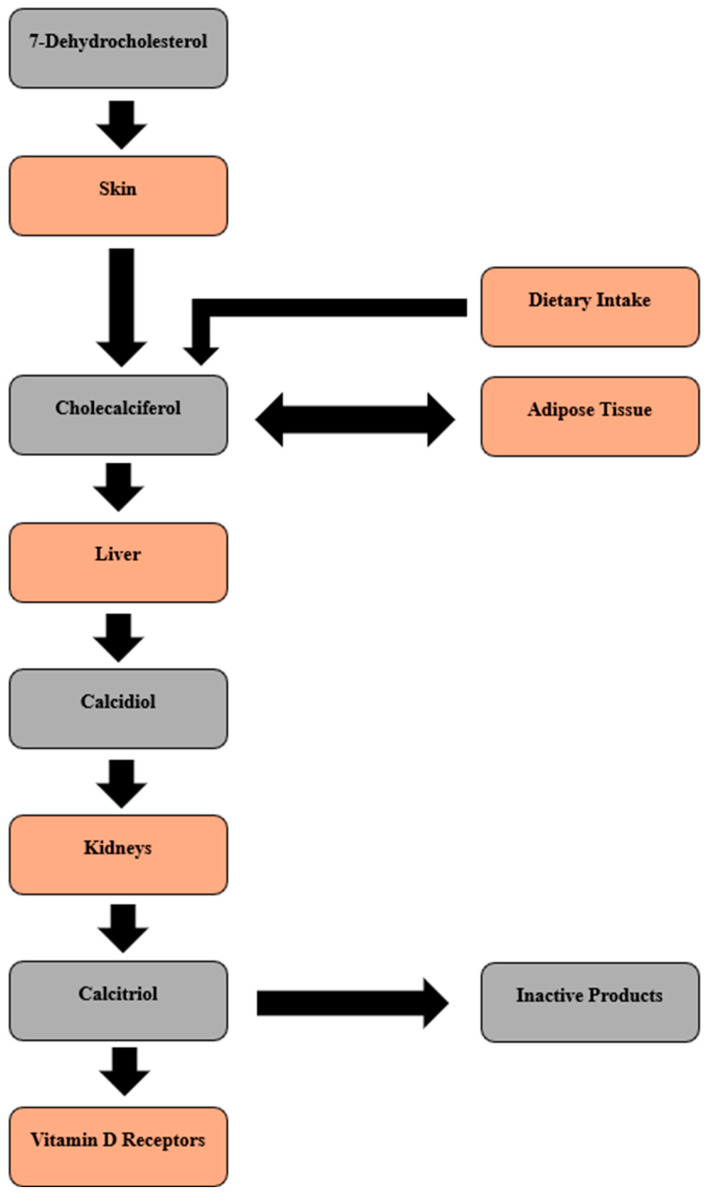
Vitamin D metabolism.

Vitamin D synthesized in the epidermis is released into systemic circulation bound to the vitamin D-binding protein (DBP) [3]. Once in the bloodstream, vitamin D is either stored in adipose tissue or converted by hepatic cytochrome P450 enzymes into calcidiol. The level of 25-hydroxyvitamin D (25(OH)D) serves as an indicator of the vitamin D status, reflecting sunlight exposure, supplementation, and dietary intake. When required, 25(OH)D is converted in the kidney to its active hormonal form, calcitriol (1,25-dihydroxyvitamin D or 1,25(OH)2D), in a process regulated by the parathyroid hormone. Excess vitamin D is converted into inactive products such as lumisterol and tachysterol, balancing cutaneous synthesis as a physiologic loop to prevent vitamin D “overdose” from sun exposure [4]. 1,25(OH)2D acts as a high-affinity ligand for the vitamin D receptor (VDR) in target tissues where it modulates the expression of vitamin D-responsive genes. VDRs are present in a wide range of tissues and cells including the small intestine, the colon, osteoblasts, T and B lymphocytes, and mononuclear cells. Moreover, VDRs are found in several major organs, including the brain, heart, skin, gonads, prostate, and breasts [5]. Figure 1 depicts vitamin D metabolism in graphic form.

### 1.2. Serum Concentrations of 25(OH)D

Vitamin D concentration is most often detected using the 25-hydroxyvitamin D (25(OH)D) radioimmunoassay. As 25(OH)D is the primary circulating metabolite of vitamin D, it is considered the most reliable marker for assessing vitamin D status. Table 1 summarizes serum 25(OH)D concentrations and their associated health status. Vitamin D deficiency is often asymptomatic, however if symptoms do appear, they are usually non-specific, such as fatigue, muscle weakness, bone pain, a depressed mood, and impaired cognitive function. Administering vitamin D at intervals, whether three times a year, weekly, or daily, can effectively maintain serum calcidiol levels across all age groups [6]. There is a large margin of safety for vitamin D oral intake, with toxicity usually observed only in patients taking doses of around 40,000 IU daily [7]. Intoxication may manifest symptoms such as headaches, a metallic taste, nephrocalcinosis, vascular calcinosis, pancreatitis, nausea, and vomiting [6,7].

### 1.3. Vitamin D Sources

#### 1.3.1. Sun Exposure

The primary natural source of vitamin D is the influence of sunlight (UVB radiation) on the skin [9]. Optimal endogenous synthesis occurs with exposure of the face, arms, hands, and legs to the sun (without sunscreen) for approximately 5 to 30 min on most days of the week [10]. However, because UV radiation is a well-known risk factor for skin cancer [11,12], numerous international and national organizations (including the WHO, FDA, CDC, and NHS) recommend the use of sunscreen with a Sun Protection Factor (SPF) of 15 or above to mitigate the risk. Interestingly, recent evidence suggests that sunscreen can effectively prevent harmful UV exposure without significantly impairing vitamin D synthesis [13].

Several additional factors influence the effectiveness of vitamin D production through sun exposure, including latitude, altitude, season, skin type, and age among others. Studies have shown that at latitudes of ±40°, the skin produces minimal or no vitamin D during the fall and winter months due to insufficient UV exposure [14,15]. In contrast, at lower latitudes (<25°), the seasonal differences between wintertime and summertime vitamin D levels are minimal, suggesting that increased sun exposure in winter may be unnecessary and could lead to excessive UV exposure [16]. Contrary to previous findings, a European study reported that northern Europeans exhibited higher 25(OH)D levels than southern Europeans. This finding is attributed to higher vitamin D food fortification, dietary habits, and more prevalent vitamin D supplementation. These findings emphasize that sun exposure alone is not the sole determinant of vitamin D status [17].

#### 1.3.2. Dietary Intake

Vitamin D is a fat-soluble compound naturally present in only a limited number of foods, including certain fish species (such as sardines, herring, tuna, mackerel, and salmon), egg yolks, shitake mushrooms, and liver. In many countries, common products such as milk, margarines, and breakfast cereals are routinely fortified with vitamin D. Randomized control trials (RCTs) have confirmed the safety of food fortification with vitamin D and have demonstrated corresponding increases in serum 25(OH)D concentrations [18]. Notably, a Finnish study reported a significant decline in vitamin D insufficiency among individuals not taking supplements, from 59% in 2000 to 14% in 2011. The improvement was primarily attributed to dietary modifications, particularly the increased consumption of fortified foods [19]. Primary 25(OH)D dietary sources vary across populations, likely reflecting cultural and dietary habits [20,21].

#### 1.3.3. Supplementation

Guidelines for vitamin D intake vary across countries and health organizations, reflecting both an incomplete understanding of the biological and clinical implications of vitamin D as well as the differing goals and target populations for the guidelines. The committee established by the Institute of Medicine reviewed the dietary intakes for vitamin D and calcium and determined a recommended dietary allowance (RDA)—defined as the average daily intake sufficient to meet the nutrient requirements of nearly all (97–98%) healthy individuals. The RDA for adults aged 19–70 years is set at 600 IU per day and at 800 IU per day for adults over 70 years, with a tolerable upper intake level of 4000 IU for both groups [22]. The updated Polish guidelines recommend that healthy adults aged 19 to 65 do not require cholecalciferol supplementation from May through September, provided they maintain regular sun exposure. However, supplementation with cholecalciferol in a dose of 1000–2000 IU/day is still considered safe and is recommended year-round for individuals who do not obtain sufficient sunlight. For adults aged between 65 and 75 years, a daily dose of 1000–2000 IU is recommended throughout the year, while individuals over 75 years of age are advised a dose of 2000–4000 IU/day [23]. In contrast, the Endocrine Society does not recommend routine vitamin D supplementation or routine 25(OH)D screening for adults aged 19 to 74. For individuals over 75 years, they suggest empirical vitamin D supplementation, preferring lower daily doses over less frequent higher doses [24]. Vitamin D3 is generally preferred over vitamin D2 as a supplement due to its longer half-life, greater potency, and lower cost. Cholecalciferol is more potent, leading to 2–3 times greater storage of vitamin D than ergocalciferol. Replenishing the deficiency depends on the baseline serum 25(OH)D concentration and the capacity for effective absorption. It is estimated that every 100 IU of vitamin D3 increases serum 25(OH)D by 0.7–1.0 ng/mL [25].

## 2. Materials and Methods

This review was conducted between October 2024 and May 2025. It did not require submission to or approval by an ethics committee, since it corresponded to a systematic review of the literature published in this field of research. The articles included in the current review were selected exclusively from indexed databases.

A systematic search was conducted in the PubMed and Web of Science databases to identify original empirical studies published between 2000 and 2024. The search strategy included combinations of terms related to schizophrenia, psychosis, and vitamin D. Ten free-text queries were run separately in both databases. The search terms were as follows: “vitamin d schizophrenia”, “vitamin schizophrenia”, “schizophrenia supplementation”, “psychosis vitamin d”, “psychosis vitamin”, “psychosis supplementation”, “calciferol schizophrenia”, “cholecalciferol schizophrenia”, “calciferol psychosis”, and “cholecalciferol psychosis”. The session histories and exact hit-counts were not fully retained. To ensure reproducibility, all queries were re-executed on 10 of July 2025, and the verbatim search strings, Automatic Term Mapping expansions, and current record totals were recorded. The record counts differed by ≤4%, owing to routine database updates; no study screened in December 2024 was lost.

The eligibility criteria included a clinical diagnosis of schizophrenia and a requirement to report the quantitative measurement of vitamin D status (serum 25(OH)D). Only studies with a full text published in English were considered. In cases of studies that included participants with multiple psychiatric diagnoses, only those with clearly defined and reported data for the schizophrenia diagnosis were considered. Studies on first psychotic episodes were considered only when a schizophrenia diagnosis had been made and the results were reported separately for that subgroup.

Studies that lacked full text access, were published in languages other than English, focused on diagnoses different than schizophrenia (unless schizophrenia data were reported separately), or did not report measured vitamin D levels were excluded. Non-empirical studies (e.g., reviews, meta-analyses, editorials, commentaries, and letters) and studies with no original data were also excluded. Supplementation studies were excluded if vitamin D was administered exclusively to participants with a pre-existing deficiency or insufficiency at the baseline as such designs confounded supplementation effects with baseline status, preventing the assessment of the relationship between vitamin D levels and schizophrenia across the full spectrum of vitamin D concentrations.

**Figure 2:**
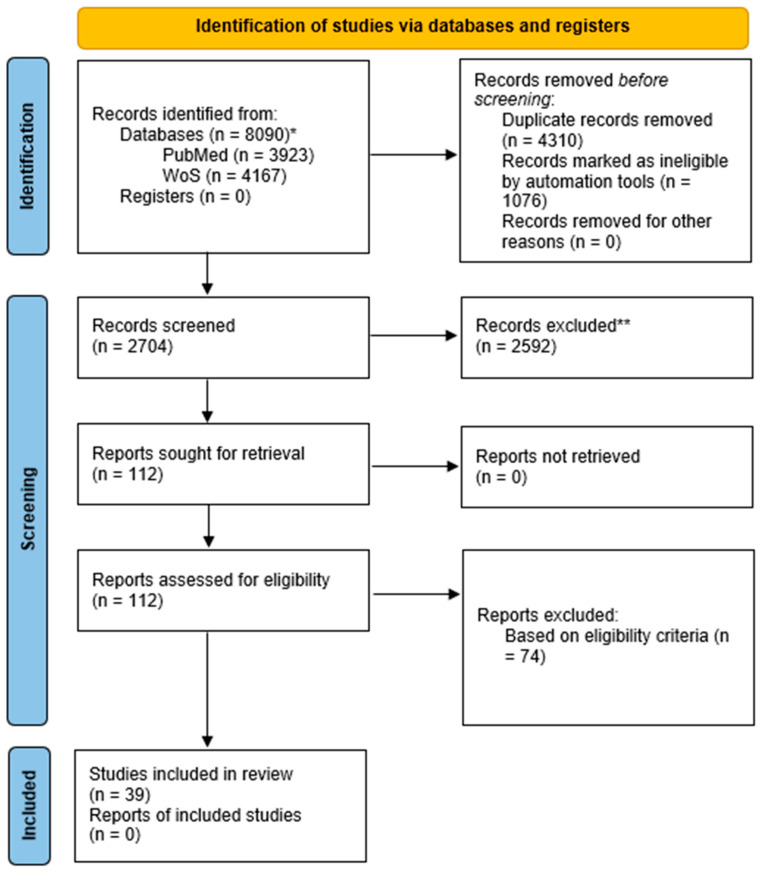
PRISMA statement for systematic review. * Counts re-run on 10 July 2025 (December 2024 figures were partly unavailable). ** Records excluded based on title and abstract.

The re-execution of records on 10 of July 2025 identified 8088 records that were imported to Papers by ReadCube. Built-in automation tools were used for removing duplicates, bringing the number of studies down to 4305. In the next step, the studies were automatically filtered, excluding those published before the year 2000, giving a total of 2704. Based on their title and abstract, a total of 103 studies were chosen for full text screening. During the manuscript revisions, 9 additional studies were identified for additional screening (access to full text was obtained), bringing the total number of studies assessed for eligibility to 112. The studies were distributed between two independent reviewers, who determined the final inclusion based on the full text. Any doubts and disagreements were addressed through discussion, and the final selection was reached by consensus among the reviewers. In total, four studies on the neonatal and maternal vitamin D of schizophrenic patients and thirty-five studies on vitamin D levels in schizophrenic patients were included in the current review (Figure 2).

In this review, the risk of bias was apprised using the Newcastle–Ottawa Scale (NOS). Each study was assessed by two reviewers (J.M., B.M) independently awarding points in each of the scales’ domains. Case–control studies were assessed with a standard form; for cross-sectional studies, NOS-xs was used [26]. Disagreements were resolved through discussion. The overall NOS and NOS-xs ratings and individual domain scores can be found in Table 2 and Table 3.

Few studies investigated comprehensively and adjusted for confounders such as BMI, sun exposure, smoking status, comorbid medical conditions, and medication adherence. Basic demographic data such as age and sex were reported. Most of the studies were conducted in a single unit (e.g., hospital/clinic), which by design restricted ethnicity. Similarly, comorbid medical conditions were often excluded by design, with some studies deciding on real-world scenarios by deliberately including potential biases while increasing external validity. A summary of the confounders investigated in each study is presented in Table 4.

In preparation of this systematic review, generative AI tools were utilized to assist with the translation and enhancement of the readability and language quality of the manuscript, as well as for refining the methodology section. All intellectual content, analysis, and interpretations presented in this work remain the original work of the authors. The use of AI tools did not influence the scientific integrity or conclusions of the review.

## 3. Results

### 3.1. Maternal Vitamin D

The first evidence linking maternal malnutrition and schizophrenia in offsprings emerged from studies of individuals born during the Dutch Hunger Winter [66]. Epidemiological observations, such as the increased prevalence of schizophrenia among individuals born in the winter months, led to the hypothesis that prenatal vitamin D deficiency may play a role in modifying the risk of schizophrenia [67]. In the early 2000s, the first study testing the hypothesis was published. The study found no significant differences in maternal 25(OH)D levels between individuals with schizophrenia and the matched controls, even when analyses were restricted to those with deficient vitamin D (below 15 ng/mL) [27]. Similarly, a large nationwide Finnish population-based case–control study found no significant association between the maternal vitamin D levels in early pregnancy (first and beginning of second trimesters) and schizophrenia risk in the offsprings [28]. Interestingly, a subgroup analysis examining Black individuals separately revealed a non-significant trend-level in the predicted direction, suggesting that race-related biological or environmental factors may influence the potential association between vitamin D and schizophrenia risk [28].

### 3.2. Neonatal Vitamin D

**Figure 3:**
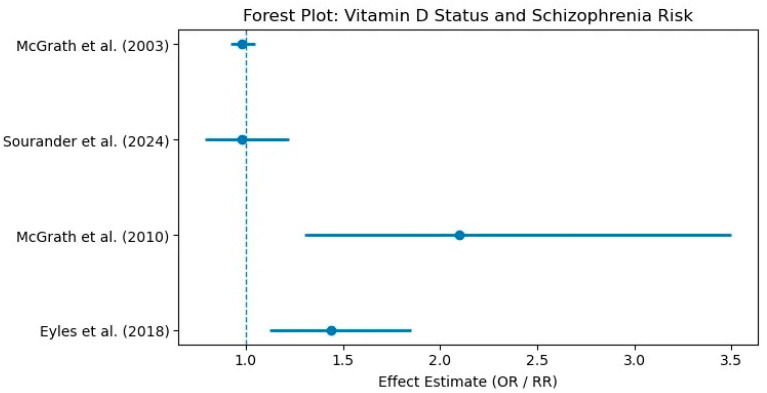
Forest plot: Vitamin D status (maternal and neonate) and schizophrenia risk [27,28,29,30].

Two studies have investigated the association between neonatal vitamin D status and the risk of developing schizophrenia later in life. Both studies measured vitamin D levels in neonatal dried blood samples, with some samples dating back to 1981, rising some concerns about potential degradation. Despite this limitation, both studies reported a significant association between neonatal vitamin D deficiency and an increased risk of schizophrenia in adulthood [29,30]. In the first published study, neonates in the lowest three quintiles of vitamin D, compared with those in the fourth quintile (25[OH]D3 levels between 40.5 and 50.9 nmol/L), had approximately a two times greater risk of developing schizophrenia. Notably, individuals in the fifth (highest) quintile also demonstrated a significantly elevated risk, suggesting a potential U-shaped relationship between vitamin D status and schizophrenia risk [29]. The second study found an increased risk of schizophrenia only among neonates in the lowest quintile (<20.4 nmol/L); no significant associations were observed in the other quintiles [30]. A summary of the studies examining the association between maternal and neonatal vitamin D concentrations and the risk of schizophrenia is presented in Table 5. The mean vitamin D levels and the effect estimates are presented in Figure 3.

### 3.3. Vitamin D Status in Population Diagnosed with Schizophrenia

**Figure 4:**
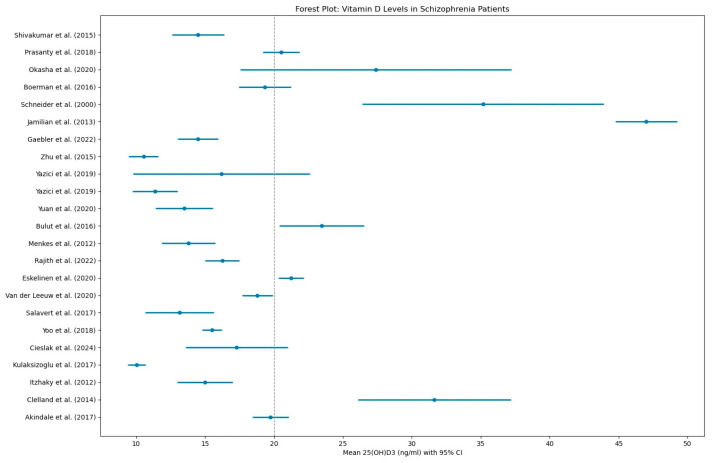
Forest plot: Vitamin D levels in schizophrenia patients [32,33,34,36,37,39,40,41,42,43,44,45,48,50,51,52,53,54,55,57,60,61,62].

The high prevalence of vitamin D insufficiency or deficiency among individuals with schizophrenia has been consistently reported in numerous publications throughout the years (Figure 4).

**Figure 5:**
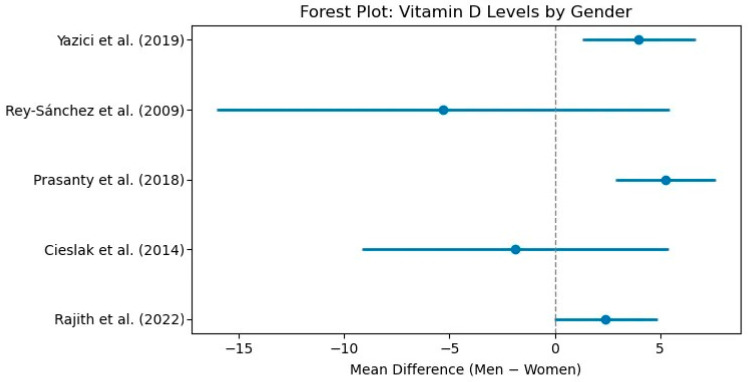
Forest plot: Vitamin levels by gender [31,37,41,52,62].

Few studies investigated the sex differences in 25(OH)D concentrations among schizophrenia patients with inconsistent results. The reported mean concentration for female participants ranged from 14.7 ± 4.8 ng/mL to 21.12 ± 11.78 ng/mL, while the values for males ranged from 15.12 ± 11.96 ng/mL to 23.64 ± 4.99 ng/mL. Some studies reported significantly lower vitamin D concentrations amongst females compared with males [31,37,52], whereas two studies reported the opposite trend, with higher vitamin D levels in females [53,56]. Other investigations found no statistically significant differences between sexes [41,48,57,62]. Figure 5 showcases the differences in vitamin D status between genders.

Of the studies included in Figure 4 sixteen reported mean vitamin D concentrations below 20 ng/mL [34,36,37,39,41,42,43,44,45,51,55,57,61,62,64], a level considered inadequate for bone and health overall. Among the studies, three found mean 25(OH)D levels that fell below the clinical deficiency threshold [34,44,64] of 12 ng/m. One study reported vitamin D levels below the deficiency threshold in specific subgroups only, such as patients treated with olanzapine, paliperidone, or amisulpride and in nonmedicated individuals. In contrast, patients treated with aripiprazole did not meet the deficiency criteria, although their mean level was still low at 13.5 ng/mL [39]. Interestingly, one study reported a mean deficiency among outpatients, but not inpatients, suggesting possible environmental or treatment-related differences between clinical settings [36]. A summary of the studies on vitamin D status in schizophrenia patients can be found in Table 6.

### 3.4. Symptoms

The severity of schizophrenia symptoms is commonly assessed using standardized clinical scales, including the Positive and Negative Syndrome Scale (PANSS), the Scale for the Assessment of Negative Symptoms (SANS), and the Scale for the Assessment of Positive Symptoms (SAPS). These tools provide objective assessments of symptom severity and are frequently utilized in scientific research. Cognitive functioning is often assessed with The Brief Assessment of Cognition in Schizophrenia (BACS).

In this review eight studies reported data on positive and negative symptoms in relation to vitamin D status, with mixed results. Five studies found no significant correlation between vitamin D and symptom severity as measured by the standardized scales [36,38,45,50,51]. Three studies reported contrasting results. One study found that lower serum vitamin D levels were associated with higher total PANSS scores [52]. Another study observed that reduced vitamin D levels correlated with both positive and negative symptoms, as well as greater overall symptom severity [40]. A third study reported that higher vitamin D concentrations in individuals with schizophrenia were linked to fewer and less severe positive and negative symptoms [42].

Only two studies investigated cognitive symptom severity. One study found a positive association between vitamin D levels and cognitive performance, specifically in processing speed (Trail Making Test Part A—TMT-A and BACS Symbol Coding) and executive functioning (Trail Making Test Part B—TMT-B and BACS Tower of London). However, after adjusting for additional cognitive variables and applying the Bonferroni correction, the association remained statistically significant only for TMT-A [55]. In contrast, Ling et al. reported no significant difference in the scores of the Repeatable Battery for the Assessment of Neuropsychological Status (RBANS) between the vitamin D sufficient and insufficient groups, suggesting no direct association between vitamin D status and overall cognitive functioning in schizophrenia patients [65]. The findings are summarized in Table 7.

### 3.5. Medication

Some studies have examined the relationship between vitamin D and the pharmacological treatment administered to patients with schizophrenia [39,41,45,63]. Rajith et al. found no significant effect of either first- or second generation antipsychotics on 25(OH)D levels [41]. Similarly, Itzhaky et al. reported no association between vitamin D concentrations and the type or combination of medications administered, including antipsychotics, antidepressants, sedatives, and mood stabilizers [45].

One study reported significantly lower vitamin D concentrations in non-medicated patients compared to healthy controls; however, the results should be interpreted cautiously due to the small sample size of the non-medicated group (*n* = 6). Interestingly, a positive correlation was observed between aripiprazole serum levels and 25(OH)D concentrations in this study, suggesting a potential interaction. No such correlation was observed in patients treated with olanzapine, paliperidone, or amisulpride [39]. In contrast, Goh et al. observed significantly lower vitamin D levels in patients receiving atypical antipsychotics (primarily olanzapine and/or risperidone) and those on combination therapy, compared with healthy controls. These associations remained significant after adjusting for covariates. However, the effects of individual antipsychotics on vitamin D levels were not assessed separately [49]. Similarly, Gaebler et al. observed a negative relationship between vitamin D levels and certain antipsychotic drug concentrations, particularly those metabolized by cytochrome P450 3A4 (CYP3A4), such as aripiprazole and quetiapine. This finding suggests that higher vitamin D levels may potentially reduce the therapeutic effect of certain antipsychotics via metabolic interactions [63].

Extrapyramidal symptoms (EPS) are a common side effect of antipsychotic treatment. They are particularly common with drugs blocking dopamine D2 receptors. Shahini et al. reported that improvements in EPS were positively associated with higher serum vitamin D levels. However, due to the small sample size and lack of stratification by antipsychotic type, definite conclusions could not be drawn [38]. The findings are summarized in Table 8.

### 3.6. Neurobiological Implications

One study reported a significant positive correlation between serum vitamin D levels and the right hippocampal gray matter volume in drug naive/free schizophrenia patients, suggesting that lower vitamin D concentrations were associated with reduced gray matter volume in this region of the brain. Notably, this association was not observed in the left hippocampus. The authors acknowledged that while the correlation was statistically significant, it did not establish a causal relationship. It pointed to a potential role of vitamin D in the pathogenesis of schizophrenia, warranting further investigation [51].

Similarly, only one study has concurrently measured brain-derived neurotrophic factor (BDNF) alongside vitamin D in patients with schizophrenia. This study found that both vitamin D and BDNF levels had significantly lower values in schizophrenia patients compared with healthy controls, and it reported a positive correlation between the two biomarkers. Given the limited research on the interplay between vitamin D and BDNF in schizophrenia, these results show a potential direction for future research [44].

### 3.7. Bone Health

Vitamin D and the parathyroid hormone (PTH) work in a regulated negative feedback loop. Lower serum 25(OH)D concentrations lead to increased PTH secretion. Four studies examined this relationship in patients with schizophrenia. Three of them reported a significant negative correlation between serum PTH and vitamin D levels [31,54,56], supporting the presence of secondary hyperparathyroidism in the context of vitamin D deficiency. In contrast, Arya et al. reported significantly lower PTH levels in patients with schizophrenia compared to healthy controls, suggesting a potential dysregulation of the feedback mechanism in this population [58].

Elevated parathyroid hormone concentrations have been associated with bone demineralization and an increased risk of fractures. Two studies specifically focused on bone mass and the skeletal status of schizophrenia patients. One study found that women with schizophrenia exhibited significantly lower bone ultrasound values compared to the matched controls, indicating a compromised skeletal status and an increased risk of fragility fractures [59]. Another study reported a greater loss of phalangeal bone mass among women treated with antipsychotics. The loss was associated with low levels of vitamin D, secondary hyperparathyroidism (elevated PTH), and increased bone turnover. The authors attributed these findings to the adverse effects of long-term antipsychotic treatment [31]. Notably, in both studies, the effects were not observed in male patients, suggesting a possible gender-related variability in the skeletal outcomes [31,59].

### 3.8. Metabolic Syndrome

Metabolic syndrome (MetS) is highly prevalent among individuals diagnosed with schizophrenia. Its etiology is multifactorial, with antipsychotic medication recognized as one of the primary contributing risk factors. While many studies report data on metabolic indicators (body mass index—BMI, triglyceride levels, and HDL cholesterol), they do not directly assess the relationship between MetS and vitamin D. Two studies examined this association. In a study by Yoo et al., 32.1% of patients met the criteria for metabolic syndrome. It was reported that vitamin D insufficiency was significantly associated with both metabolic syndrome and hypertension, even after adjusting for physical activity and dietary habits [61]. Similarly, another study reported that 42.18% of schizophrenia patients met the MetS criteria. Although lower levels of vitamin D were observed in the schizophrenia patients with metabolic syndrome compared with those without MetS, the difference did not reach statistical significance [44]. However, due to the cross-sectional character of both studies, it was not possible to establish a causal relationship between vitamin D and metabolic syndrome [44,61].

### 3.9. Influence of Sun Exposure on Vitamin D Concentration

Sun exposure is one of the primary determinants of vitamin D concentration in the general population. Several studies have examined the daily sun exposure of individuals with schizophrenia in comparison with healthy controls. Most reported no significant differences in sun exposure between the groups [45], nor did they find a relationship between serum 25(OH)D and the number of hours spent in the sun [41,43,47].

In contrast, Lally et al. reported a direct positive association between vitamin D and sun exposure in schizophrenia patients [64]. Similarly, Azar et al. found that both sun exposure and being in an intermediate to high socioeconomic group significantly increased the likelihood of having an increased level of vitamin D compared to individuals from low socioeconomic groups [46].

Additionally, one study focused on urbanicity at birth as a potential factor influencing vitamin D status. The findings revealed a negative association between urbanicity at birth and vitamin D concentration, suggesting that low 25(OH)D in schizophrenia might be mediated by early environmental risk factors [42].

### 3.10. Influence of Blood Markers on Vitamin D

Several studies alongside vitamin D have investigated other biochemical and nutritional markers including CRP [34,58]; folic acid; vitamins B12 [37], A, and E [39]; homocysteine [36,38]; and proline levels [48].

Elevated mean CRP levels and reduced vitamin D concentrations were commonly reported in schizophrenia patients, in contrast to the matched controls. Notably, one study found that individuals with both elevated CRP and sufficient vitamin D had a significantly lower incidence of schizophrenia compared with those with insufficient vitamin D. Participants with an elevated CRP and reduced 25(OH)D levels had the highest prevalence of schizophrenia, while the lowest prevalence was seen in participants with reduced CRP and an elevated 25(OH)D [34]. Similarly, Arya et al. reported elevated CRP levels in schizophrenia patients, with chronic schizophrenia patients exhibiting a significantly higher CRP and lower vitamin D levels than those experiencing first-episode psychosis [58]. Schizophrenia patients exhibited a higher incidence of folic acid and vitamin B12 deficiency compared to healthy controls [37], and another study found lower concentrations of vitamin E, suggesting the presence of broader multivitamin deficiencies in this population [39].

Homocysteine is an amino acid linked to the pathophysiology of psychiatric disorders. Yazici et al. compared homocysteine levels in the acute phase and the remission of schizophrenia with those of healthy controls. The study found significantly elevated homocysteine levels in patients during acute psychotic episodes compared with both remitted patients and the healthy controls. However, no significant relationship was established between homocysteine and vitamin D levels [36]. Shahini et al. reported similar findings with elevated homocysteine and low vitamin D concentrations in schizophrenia patients, but no significant relationship between the two markers [38].

Clelland et al. investigated the relationship between vitamin D and proline metabolism. Hyperprolinemia, a condition characterized by elevated amino acid proline due to impaired degradation was found to be significantly more common in individuals with vitamin D insufficiency. Moreover, hyperprolinemia was significantly associated with schizophrenia, consistent with previous research [48].

### 3.11. Other Mental Disorders

Majority of the studies comparing schizophrenia patients to those with other psychotic disorders, such as depression [32,33,54], substance use disorder [54] or bipolar disorder [53,64], found that the difference in vitamin D levels, when present, was not statistically significant [32,33,37,43,53,54]. However, a few exceptions were reported. For instance, Itzhaky et al. and Menkes et al. found that patients with schizophrenia had significantly lower serum vitamin D levels compared to patients with depression [45,57] and mania [57]. Additionally, Yazici et al. reported significantly lower 25(OH)D concentrations when compared with patients with substance use disorder [37].

When comparing the schizophrenia group with the healthy controls, the difference was more consistently pronounced across numerous publications [32,33,34,35,37,38,42,43,45,53,59]. One study reported a mean difference of over 10 ng/mL in serum 25(OH)D concentrations [32]. Another study found this difference among female patients only [31]. Notably, Boerman et al. reported that vitamin D deficiency was 4.7 times more common among outpatients with bipolar disorder, schizophrenia, or schizoaffective disorder compared with the Dutch general population [53]. Only a few studies failed to find a statistically significant difference in vitamin D status between schizophrenia patients and either the other psychiatric groups or the healthy controls [36,40,41,46,58].

## 4. Discussion

Pregnant women and newborns are widely recognized as populations at increased risk for vitamin D deficiency. The global prevalence of 25(OH)D concentrations below 20 ng/mL have been reported in 54% of pregnant women and 75% of newborns. A strong correlation between maternal and neonatal vitamin D levels has been well established [68].

Given the known association between insufficient vitamin D and negative health outcomes, several studies have explored its potential implications for mental health—schizophrenia in particular. Interestingly, no significant relationship has been observed between maternal 25(OH)D concentrations during pregnancy and schizophrenia diagnoses in offsprings [27,28]. However, an association was identified when neonatal vitamin D levels were considered [29,30]. Nevertheless, all the studies have measured vitamin D status at a single time point and have not accounted for many confounding factors known to influence 25(OH)D levels. As a result, it is impossible to draw a definite conclusion on a causal relationship. It is also possible that the period of susceptibility to schizophrenia extends beyond the prenatal period. Supporting this, a Finnish cohort study found that the absence of vitamin D supplementation during the first year of life significantly increased the risk of schizophrenia in males [69]. These findings suggest that both the timing and duration of vitamin D exposure—as well as potential confounders—must be considered in future research investigating the role of vitamin D in the pathogenesis of schizophrenia.

Individuals born during the winter months tend to exhibit lower vitamin D concentrations than those born in other seasons. This suggests that the season of birth may have a long-term impact on vitamin D status later in life [70]. A recent meta-analysis concluded that winter births were associated with a slightly increased risk of schizophrenia, whereas summer births were associated with a reduced risk [71]. In support of this, another study found that a low perinatal sunshine duration was associated with an earlier age of schizophrenia diagnosis [72]. Despite these findings, the current review found that daily exposure to sunshine in adulthood was comparable between schizophrenia patients and healthy controls, suggesting that early-life or developmental exposures may be more critical in modulating long-term schizophrenia risk.

A meta-analysis published in 2023 estimated that the global prevalence of vitamin D deficiency (serum 25(OH)D < 12 ng/mL) was at 15.7% during the period from 2000 to 2022 [73]. Schizophrenia patients exhibit a high prevalence of below optimal vitamin D concentrations, regardless of illness phase (acute or remission). Among the studies included in this review, the reported rates of vitamin D deficiency ranged from 9% to 83%, while insufficiency ranged from 14% to 78%. Notably, one study reported a 4.7 times higher prevalence of vitamin D deficiency among individuals with psychotic disorders compared with the general Dutch population [53].

Clinical trials have demonstrated that vitamin D supplementation in schizophrenia patients, whether daily, weekly, biweekly, or monthly, effectively increases 25(OH)D levels. Additionally, some of the studies reported an enhanced cognitive performance [74], an improved attention span [75], and a reduction in the PANSS score when vitamin D and probiotics were co-supplemented. These findings suggest that low vitamin D may negatively affect the course of the disease and symptom severity, highlighting the potential role of supplementation in this population [76].

Many studies investigating vitamin D status among psychiatric patients often do not differentiate between specific diagnosis (especially psychotic disorders) and treat them as a single group [77]. The current findings support the observation that low vitamin D levels are common among psychiatric patients and do not allow for a reliable distinction between mental illnesses. However, a limited number of studies reported the difference between specific mental disorders. Notably, significantly different vitamin D levels were observed in patients with schizophrenia compared with those with depression [39,50] and a substance use disorder [37], suggesting that, under certain conditions, diagnostic distinctions in vitamin D status may emerge.

Vitamin D metabolism involves several cytochrome P450 enzymes such as CYP2R1, CYP27A1, CYP3A4, and CYP2D25. Among these, CYP3A4 is particularly notable, as it is responsible for the metabolism of approximately 50% of all known drugs. Given its role in both vitamin D and drug metabolism, questions were raised about potential interactions when co-administered [78]. This concern was supported by findings from Gaebler et al., who reported an association of higher vitamin D levels with reduced antipsychotics levels, particularly the ones primarily metabolized by CYP3A4. Reduced vitamin D concentrations might in turn cause slower drug elimination, increasing the possibility of adverse effects [63].

The association between schizophrenia and structural abnormalities of the hippocampus, such as volume reduction and incomplete inversion, is well established [79]. In this review, only one study was identified that directly examined the relationship between vitamin D and brain volume in individuals with schizophrenia. This study reported that higher levels of vitamin D were associated with increased right hippocampal gray matter volume [51]. These findings are notable given that the hippocampus has been shown to express functional vitamin D receptors (VDRs) in rodent models, and vitamin D has been demonstrated to influence neuronal proliferation in rat brain cells [80].

Additionally, two studies examined brain volume in broadly defined psychotic disorders. One reported a positive association between serum vitamin D concentrations and peripheral gray matter volume, although no relationship was observed with ventricular volume [81]. Another study found that lower vitamin D was associated with reduced intracranial volume [82]. These results suggest a potential structural impact of vitamin D on brain development and morphology, which warrants further investigation in psychosis and schizophrenia-specific populations.

Possible neurobiological pathways have been suggested to explain the relationship between vitamin D and schizophrenia pathophysiology [34]. The brain-derived neurotrophic factor (BDNF) has been investigated due to its crucial role in the development of the central nervous system and in maintaining neuronal connectivity. Altered BDNF expression has been implicated in the pathogenesis of schizophrenia [83]. One cross-sectional study found a positive correlation between vitamin D and BDNF levels, with both displaying significantly lower levels in patients with schizophrenia compared to healthy controls. Given that BDNF gene expression is modulated by neuronal activity involving calcium signaling, and vitamin D influences intracellular calcium transport through non-genomic mechanisms, this interaction appears biologically plausible [84]. However, as these findings stem from a single study, further research is required to validate and clarify the nature of this relationship [44]. Psychotic disorders are associated with a higher metabolic risk, which is mediated by lifestyle factors (such as poor dietary choices, physical inactivity, and a high prevalence of smoking), genetic predispositions, and the side effects of antipsychotic medication [85]. The widespread use of second-generation antipsychotics has led to an increase in side effects such as obesity, insulin resistance, and diabetes. The incidence of MetS in patients with schizophrenia ranges between 5.6% and 63%. The mechanism responsible for these side effects are not clearly defined, with studies on lifestyle modifications yielding mixed results [85]. Since vitamin D has properties that could potentially reduce metabolic side effects, further studies on this topic are warranted [85]. The levels of circulating vitamin D can be reduced in obese individuals because it is fat soluble and can be stored in adipose tissue. Among the included studies, the prevalence of metabolic syndrome ranged from 32% to 42%. While vitamin D deficits appear to coexist with MetS, the association between the two in schizophrenia patients needs further examination due to mixed results [44,61,85].

Peak bone mass is attained in the third decade of life, with age-related bone loss beginning in the fourth decade. In women, this process is accelerated even up to 10 years post-menopause due to hormonal changes. Prolonged low vitamin D status can lead to bone demineralization, increasing the risk of fractures. As a result of secondary hyperparathyroidism, osteomalacia and osteoporosis can develop [86]. Since patients with schizophrenia tend to have lower vitamin D levels than the healthy population, studies showcasing these effects are important for better understanding and providing specialized care for this population. Research investigating PTH in schizophrenia has shown that with low vitamin D and high PTH levels, this population is at risk of developing secondary hyperparathyroidism [58]. Studies focusing on bone mass found that women with schizophrenia exhibited lower bone mass than healthy controls, indicating a higher risk of fractures [31,59].

People diagnosed with schizophrenia often have poor dietary habits, including the increased consumption of sodium, sugar, and processed foods. These dietary choices are linked to obesity, metabolic syndrome, and higher mortality rates. Nutritional deficiencies are recognized as possible risk factors for the development of mental disorders. Supplementation with vitamins has been associated with symptom reduction and the improvement of neurological deficits related to schizophrenia. Deficiencies or low levels of antioxidant vitamins such as C, E, and beta carotene have been reported to cause oxidative stress [87]. Schizophrenic patients seem to suffer from multiple vitamin deficiencies, including vitamins A, E, and group B, in addition to vitamin D [37,39,58]. Vitamin B12 and folic acid are known to affect the nervous system in numerous ways, with low levels affecting vision and cognition. Elevated homocysteine levels [36,38], often observed among psychotic patients, can contribute to cognitive decline and vascular damage. Interestingly, supplementation with folic acid and group B vitamins reduces homocysteine concentrations [88]. However, no correlation between these findings and vitamin D has been established. High CRPs have been previously reported in studies on schizophrenia biomarkers. A meta-analysis suggested that increased high-sensitivity CRP might be a risk factor for hypovitaminosis D [89], consistent with the outcomes reported in this review [34].

While this review primarily focuses on the clinical and epidemiological associations between vitamin D deficiency and schizophrenia, it is important to note that the precise molecular and biochemical mechanisms underlying this relationship remain insufficiently understood. Although some hypotheses have been proposed, definitive mechanistic pathways have not been fully understood. Further research is needed as it may provide deeper insight into potential therapeutic targets.

Current evidence does not support the universal screening of serum vitamin D levels or empirical supplementation for the prevention or management of schizophrenia. However, monitoring vitamin D status may be warranted in high-risk individuals or institutionalized populations. We emphasize that further interventional research is necessary before definitive clinical recommendations can be made.

## 5. Conclusions

This review highlights the high prevalence of vitamin D deficiency in the population of patients diagnosed with schizophrenia. The studies showcase the different aspects of the deficiency and its correlated symptoms, both psychiatric and somatic.

Despite the high number of studies, notable gaps remain in our understanding of vitamin D influence on the course of schizophrenia. Future research is needed to fill in the gaps, especially on the molecular and enzymatic level. As vitamin D is easy to supplement and measure the blood concentration of, a better understanding of its possible involvement in the psychopathology of schizophrenia could affect future treatment outcomes.

## Figures and Tables

**Table 1:** Serum 25-Hydroxyvitamin D [25(OH)D] Concentrations and Their Associated Health Status [8].

Scheme 25. Hydroxyvitamin D [25(OH)D] Concentration (nmol/L)	Serum 25-Hydroxyvitamin D [25(OH)D] Concentration (ng/mL)	Health Status
<30	6 to <12	Vitamin D Deficiency
>30 to <50	12 to <20	Vitamin D Insufficiency—considered inadequate for bone and overall health in healthy individuals
≥50	≥20	Generally considered adequate for bone and overall health in healthy individuals

**Table 2:** Newcastle–Ottawa Scale for case–control studies.

Study	D1	D2	D3	Overall
McGrath et al. [27]	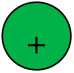	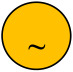	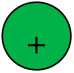	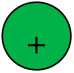
Sourander et al. [28]	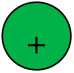	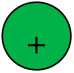	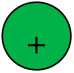	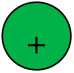
McGrath et al. [29]	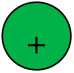	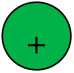	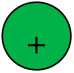	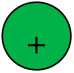
Eyles et al. [30]	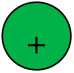	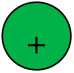	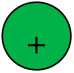	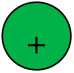
Rey-Sanchez et al. (2009) [31]	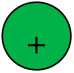	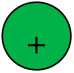	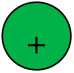	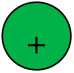
Okasha et al. (2020) [32]	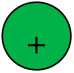	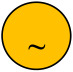	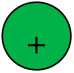	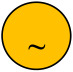
Jamilian et al. (2013) [33]	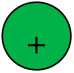	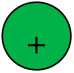	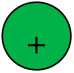	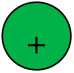
Zhu et al. (2015) [34]	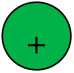	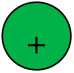	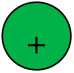	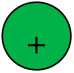
Ghosh et al. (2023) [35]	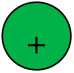	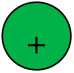	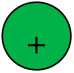	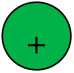
Yazici et al. (2019) [36]	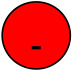	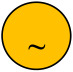	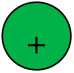	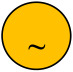
Yazici et al. (2019) [37]	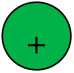	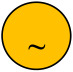	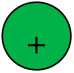	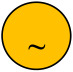
Shahini et al. (2022) [38]	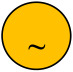	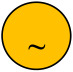	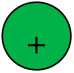	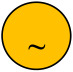
Yuan et al. (2020) [39]	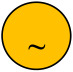	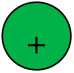	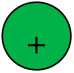	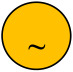
Bulut et al. (2016) [40]	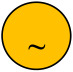	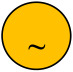	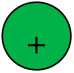	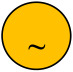
Rajith et al. 2022 [41]	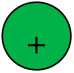	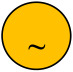	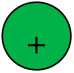	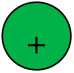
Van der Leeuw et al. (2020) [42]	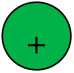	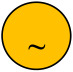	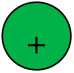	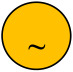
Salavert et al. (2017) [43]	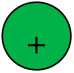	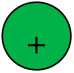	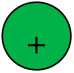	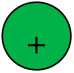
Kulaksizoglu et al. (2017) [44]	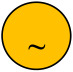	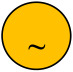	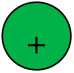	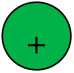
Itzhaky et al. (2012) [45]	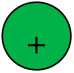	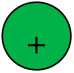	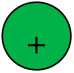	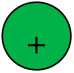
Azar et al. (2017) [46]	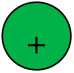	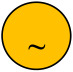	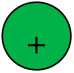	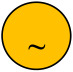
Yüksel et al. (2014) [47]	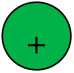	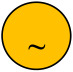	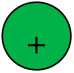	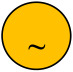
Clelland et al. (2014) [48]	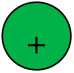	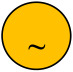	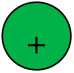	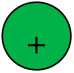
Goh et al. (2024) [49]	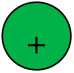	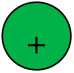	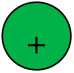	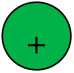
Akinlade et al. (2017) [50]	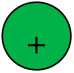	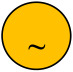	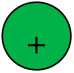	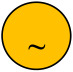

D1—Bias due to selection: 3–4 (Low); 2 (Some Concerns); 0–1 (High). D2—Bias due to comparability: 2 (Low); 1 (Some concerns); 0 (High). D3—Bias due to outcome: 2–3 (Low); 1 (Some Concerns); 0 (High). 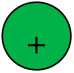 Low, 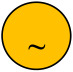 Some Concerns, 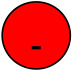 High.

**Table 3:** Newcastle–Ottawa Scale (NOS-xs) adapted for cross-sectional studies.

Study	D1	D2	D3	Overall
Shivakumar et al. (2015) [51]	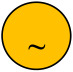	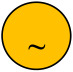	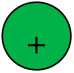	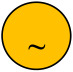
Prasanty et al. (2018) [52]	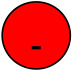	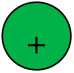	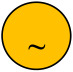	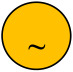
Boerman et al. (2016) [53]	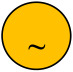	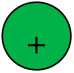	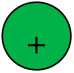	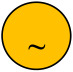
Schneider et al. (2000) [54]	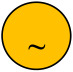	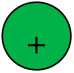	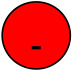	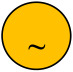
Gaebler et al. (2022) [55]	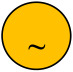	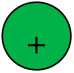	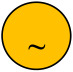	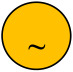
Humble et al. (2010) [56]	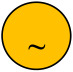	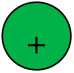	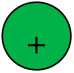	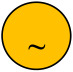
Menkes et al. (2012) [57]	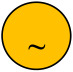	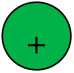	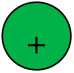	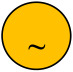
Arya et al. (2023) [58]	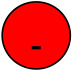	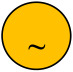	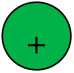	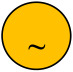
Partti et al. (2010) [59]	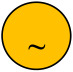	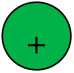	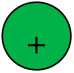	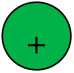
Eskelinen at al. (2020) [60]	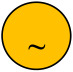	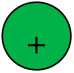	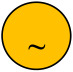	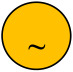
Yoo et al. (2018) [61]	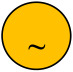	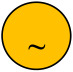	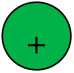	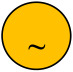
Cieslak et al. (2014) [62]	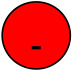	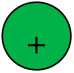	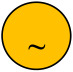	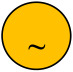
Gaebler et al. (2022) [63]	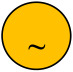	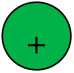	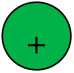	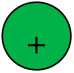
Lally et al. (2016) [64]	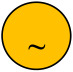	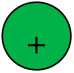	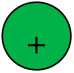	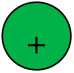
Ling et al. (2023) [65]	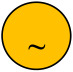	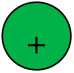	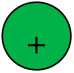	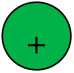

D1—Bias due to study sample selection: 2 (Low); 1 (Some Concerns); 0 (High). D2—Bias due to assessment of exposure and outcome: 3–4 (Low); 2 (Some Concerns); 0–1 (High). D3—Bias due to confounding factors: 2–3 (Low); 1 (Some Concerns); 0 (High). 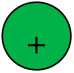 Low, 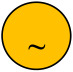 Some Concerns, 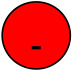 High.

**Table 4:** Summary of the confounders.

No.	Study	BMI	Sun Exposure	Ethnicity	Smoking	Comorbidities	Medication Adherence	Other Notes
1	Rey-Sanchez et al. (2009) [31]	Assessed but not included in regression models	Not assessed	Spanish	Not adjusted	Individuals with low-trauma fractures and other causes of hyperprolactinemia were excluded	Not adjusted for	Controlled for age
2	Shivakumar et al. (2015) [51]	Not assessed	Not assessed	Indian	No smoking 24 h prior to blood draw	Substance abuse (excluding nicotine) excluded	Drug naïve/free subjects	Controlled for age, years of education, and total intracranial volume (in VBM analyses).
3	Prasanty et al. (2018) [52]	Assessed but not included in regression models	30 min daily mid-day sunlight exposure prior to blood draw	Batak tribe	Not assessed	General medical/psychiatric comorbidities were excluded at enrollment—not adjusted for	Fixed dose of risperidone—not adjusted for	Key confounding factors were explicitly controlled at the design level and during statistical stratification
4	Okasha et al. (2020) [32]	Not assessed	Not assessed	Egyptian	Not assessed	General medical/psychiatric comorbidities were excluded at enrollment—not adjusted for	Not adjusted for	Controlled for age, gender, and social standard (matched)
5	Boerman et al. (2016) [53]	Not assessed	Adjusted for season of sampling	Dutch (they noted non-Western immigrants at risk)	Not assessed	General medical/psychiatric comorbidities were excluded at enrollment—not adjusted for	Not assessed	Adjusted for diagnosis, ethnicity, season of sampling
6	Schneider et al. (2000) [54]	Not assessed	Not assessed	No information on ethnicity (German sample)	Not assessed	Patients with renal or hepatic insufficiency were excluded—not adjusted for	Not assessed	Stratified comparisons and subgroup testing
7	Jamilian et al. (2013) [33]	Not assessed	Not assessed	Iranian	Not assessed	General medical comorbidities were excluded at enrollment—not adjusted for	Not assessed	General linear models (covariates included: sex, medical history, history of psychiatric hospitalization, substance abuse, education level, marital status, income level, employment status, and family history of psychiatric disorder)
8	Gaebler et al. (2022) [55]	Not assessed	Not assessed	No information on ethnicity (German sample)	Not assessed	Not assessed	Not assessed	The study adjusted for anticholinergic drug burden and mutually adjusted cognitive scores in multivariate analysis
9	Zhu et al. (2015) [34]	Adjusted for	Adjusted for season of blood draw (winter–spring vs. summer–autumn)	Chinese	Not assessed	Not assessed	Not assessed	Adjusted for age, sex, residence (urban vs. rural), BMI, education (<12 vs. ≥12 years), monthly income, and season of blood draw
10	Ghosh et al. (2023) [35]	Assessed, not adjusted for	Not assessed	Indian	Not assessed	General medical comorbidities were excluded at enrollment—not adjusted for	Drug free subjects	Controlled for confounding through design features like age/sex matching
11	Yazici et al. (2019) [36]	Not assessed	Seasonality of blood draw	Turkish	Not assessed	Not assessed	Chlorpromazine-equivalent antipsychotic dose	Controlled for age, sex, PANSS/CGI/GAF scores, inpatient vs. outpatient (relapse vs. remission), and Vitamin B_12_ and folate status
12	Yazici et al. (2019) [37]	Not assessed	Not assessed	Turkish	Not assessed	Not assessed	Not assessed	No variables were adjusted for the statistical models, and no regression or multivariate modeling was performed
13	Shahini et al. (2022) [38]	Not assessed	Not assessed	Iranian	Not assessed	Not assessed	Not assessed	Matched for age and sex (no analytic adjustment was conducted)
14	Yuan et al. (2020) [39]	Assessed, not adjusted for	Not assessed	Chinese	Not assessed	Not assessed	Assessed, but not adjusted for	Controlled for age, sex, albumin, bilirubin, triglyceride, and cholesterol
15	Bulut et al. (2016) [40]	Assessed, not adjusted for	Not assessed	Turkish	Self-reported smoking history	Excluded comorbid Axis I disorder, an organic/medical condition, and use of medications that affect vitamin D level	Not assessed	Design-based adjustments
16	Humble et al. (2010) [56]	Not assessed	Adjusted for season of blood draw	Self-reported (Northern vs. Southern origin)	Not assessed	Comorbid diagnoses present but main diagnosis used for analysis	Comorbid psychiatric diagnoses are present, but only primary diagnosis was entered into the analysis.	Ethnicity, age, sex, and diagnosis group were examined in relation to 25-OHD and iPTH levels via stratified comparisons
17	Menkes et al. (2012) [57]	Not assessed	Not assessed	Maori vs. Pākehā	Not assessed	General psychiatric outpatients	Patients represented a “real-world” outpatient population (no exclusions)	Results stratified by ethnicity (Maori vs. non-Maori), diagnosis, sex, and age
18	Arya et al. (2023) [58]	Not assessed	Not assessed	Iranian	Collected information on smoking history	Patients with any acute or chronic illness known to affect vitamin D, calcium, PTH, or CRP were excluded	Excluded any acute or chronic illness known to affect vitamin D, calcium, PTH, or CRP	Results stratified by diagnosis, first episode psychosis vs. chronic psychosis, sex, education level, and marital status
19	Partti et al. (2010) [59]	Adjusted for	Not assessed	Finnish	Adjusted for smoking status	Excluded patients on osteoporosis medication	Adjusted for current antipsychotic/mood-stabilizer use	Broadband ultrasound attenuation (BUA) and Speed of Sound (SOS), standardized by age/sex (Z-scores); women: current oral estrogen-use and menstruation status; men: highest educational level and weekly alcohol consumption
20	Rajith et al. 2022 [41]	Recorded and analyzed	Collected via self-report (hours/day); categorized as <1 h, 1–3 h, >3 h	Indian	Assessed	Excluded those with metabolic, hepatic, and renal disorders, as well as intellectual disability or substance dependence (other than nicotine)	Patients in remission with a minimum of 6 months on treatment (adherence not assessed)	The study controlled for potential confounders through inclusion/exclusion criteria and matching on age and sex
21	Eskelinen at al. (2020) [60]	Obesity status used in subgroup analyses (no mean BMI given)	Not assessed	Finnish	Not assessed	Not assessed	Not assessed	Not assessed through interaction terms or stratified models, but subgroup results for obesity and clozapine use were explored descriptively
22	Van der Leeuw et al. (2020) [42]	Not assessed	Adjusted for season of blood draw	Dutch (Caucasian) vs. non-Caucasian	Not assessed	Not assessed	Not assessed	Adjusted for age and sex; assessed PANSS scores
23	Salavert et al. (2017) [43]	Not assessed	Assessed daily sun exposure (suitable: ≥30 min/day); season of birth included as regressor	European	Not assessed	Excluded major medical/neurological disease and recent substance use	Antipsychotic-naïve at baseline	Adjusted for sex, season of birth, calcemia, Ca-intake, and sun exposure (multiple linear regression)
24	Yoo et al. (2018) [61]	Assessed, not adjusted for	Not assessed	Korean	Not assessed	Excluded active substance use	Not assessed	Adjusted for physical activity (IPAQ) and dietary habit scores
25	Cieslak et al. (2024) [62]	Not assessed	Not assessed	Not assessed	Not assessed	Not assessed	All stable on antipsychotics (no med changes ≥1 month)	Controlled for age and sex
26	Kulaksizoglu et al. (2017) [44]	Assessed, not adjusted for	Not assessed	Turkish	Assessed, not adjusted for	Excluded chronic diabetes, cardiovascular disease and hypertension	Not assessed	The study did not adjust for potential confounders in statistical modeling
27	Gaebler et al. (2022) [63]	Not assessed	Not assessed	No information on ethnicity (German sample)	Not assessed	Not assessed	Normalized for antipsychotic dose and explicitly modeled drug identity/CYP3A4-dependence	The study adjusted for analytic variables (metabolism modeling and drug identity)
28	Lally et al. (2016) [64]	Mean BMI not reported; waist circumference measured and correlated	Adjusted for season of draw	UK sample (Black African/Caribbean vs. White noted)	Assessed, not adjusted for	Excluded other psychiatric disorders	Not assessed	Adjusted for age and sex, ethnicity, and season of sampling
29	Itzhaky et al. (2012) [45]	Not assessed	Not assessed	Israeli	Not assessed	No physical health or comorbidity exclusions were imposed	Assessed, not adjusted for	Confounding assessed via group stratification and comparisons
30	Azar et al. (2017) [46]	Not assessed	Weekly hours of sun exposure (adjusted for)	Lebanese	Assessed, not adjusted for	Excluded any metabolic disease known to affect serum vitamin D concentrations	Assessed, not adjusted for	Multivariate logistic: socioeconomic level, sun exposure, and MRSS score
31	Yüksel et al. (2014) [47]	Not assessed	Daily duration of sun exposure (self-report) (not adjusted for)	Turkish	Assessed, not adjusted for	Excluded substance dependence, organic mental disorders, learning disabilities, and metabolic diseases affecting vitamin D	On antipsychotics (remission vs. acute groups)	Controlled through study design (matching on age and sex; standardized season of recruitment)
32	Clelland et al. (2014) [48]	Not assessed	Season of blood draw (adjusted for)	African-American, Caucasian, and Hispanic (matched)	Not assessed	Excluded organic disorders and substance dependence	Not assessed	Adjusted for education level, ethnicity, and season
33	Ling et al. (2023) [65]	Not assessed	Not assessed	Chinese	Not assessed	None	Not assessed	Adjusted for age, sex, education, and disease duration
34	Goh et al. (2024) [49]	WHO Asian cut-offs for subgroup analyses	Not assessed	Malaysian	Adjusted for	Excluded other psychoses and substance abuse	Stratified analyses by drug class	Adjusted for age, sex, ethnicity, and smoking status
35	Akindale et al. (2017) [50]	Assessed	Not assessed	No information on ethnicity (Nigerian sample)	Not assessed	Excluded patients with severe/unstable medical comorbidities	Not assessed	Confounding controlled through group design and mean comparisons

**Table 5:** Maternal and neonatal vitamin D in schizophrenia.

Study	Year	Study Design	*n*: Cases/Controls	Samples Collection	Diagnosis	Results
Maternal Vitamin D						
McGrath et al. [27]	2003	Case–control	Cases: 26 Controls: 51	Third trimester	DSM-IV	There was no significant difference in third trimester maternal vitamin D in the entire sample; OR 0.98 (95% CI 0.92–1.05).
Sourander et al. [28]	2024	Case–control	Cases: 1145 Controls: 1145	First and early second trimester	ICD-9 ICD-10	Maternal vitamin D levels in early pregnancy were not associated with offspring schizophrenia in unadjusted (OR 0.96, 95% CI 0.78–1.17, *p* = 0.69) or adjusted analyses (OR 0.98, 95% CI 0.79–1.22, *p* = 0.89).
Neonatal Vitamin D						
McGrath et al. [29]	2010	Case–control	Cases: 424 Controls: 424	Dried blood spots from newborns	ICD-10	Both low and high concentrations of neonatal vitamin D are associated with an increased risk of schizophrenia. Compared with the fourth quintile, neonates in the lowest quintile had a RR of 2.1 (95% CI, 1.3–3.5) while those in the second and third quintiles had a RR of 2.0 (95% CI, 1.3–3.2) and 2.1 (95% CI, 1.3–3.4), respectively. The highest quintile also had a significantly increased RR of 1.71 (95% CI, 1.04–2.8).
Eyles et al. [30]	2018	Case–control	Cases: 1301 Controls: 1301	Dried blood spots from newborns	ICD-10	Compared with the reference (fourth) quintile, those in the lowest quintile (<20.4 nmol/L) had a significantly increased risk of schizophrenia (IRR = 1.44, 95%CI: 1.12–1.85).

**Table 6:** Summary of studies on vitamin D in schizophrenia patients included in the review.

Study	Study Design	Diagnosis	*n*: Cases/Controls	Sex Distribution in Study Group	Age (Mean ± SD)	Antipsychotic Medication	Vitamin D—25(OH)D3 (ng/mL) (Mean ± SD)	Results
Rey-Sanchez et al. (2009) [31]	Cross-sectional; Case–control	DSM-IV	Cases: 73 Controls: 73	Female: 25 Male: 48	Female: 59.84 ± 17.01 Male: 61.89 ± 12.95	Yes	Female: 20.42 ± 26.05 Male: 15.12 ± 11.96	A total of 74.1% of women and 69.6% of men had vitamin D levels below 15 ng/mL. There was a significant negative correlation between vitamin D and PTH levels in both men and women (*p* < 0.0001).
Shivakumar et al. (2015) [51]	Cross-sectional	DSM-IV	Cases: 35	Female: 15 Male: 20	32.14 ± 6.6	No	14.5 ± 5.7	A total of 97% percent of the schizophrenia patients had suboptimal levels of vitamin D (83% deficiency). A significant positive correlation between vitamin D levels and the right hippocampal gray matter volume was found (*p* = 0.002). Serum vitamin D did not correlate significantly with symptom scores.
Prasanty et al. (2018) [52]	Cross-sectional	ICD-10	Cases: 54	Female: 19 Male: 26	Female: 29.684 ± 5.478 Male: 31.167 ± 8.241	Yes	Female: 18.400 ± 3.877 Male: 23.639 ± 4.990	There was a significant difference between male and female serum levels (*p* < 0.05). The lower the serum levels of vitamin D, the higher the total score of the PANSS.
Okasha et al. (2020) [32]	Cross-sectional; Case–control	DSM-IV	Cases: 20 Controls: 20	N/A	31 ± 8	Yes	27.4 ± 27.5	A total of 80% of patients with schizophrenia had below normal vitamin D levels. Schizophrenia patients had lower vitamin D levels than control group but higher levels than patients with major depressive disorder (MDD). The difference between schizophrenia and the MDD groups was not statistically significant (*p* = 0.298).
Boerman et al. (2016) [53]	Cross-sectional	DSM-IV	Cases: 149	N/A	N/A	Yes	Female: 21.12 ± 11.78 Male: 17.55 ± 9.75	Vitamin D levels were deficient in 34.7% of the patients with schizophrenia or schizoaffective disorder. There was no significant difference between bipolar disorder and schizophrenia or schizoaffective disorder. Vitamin D deficiency was 4.7 times more common among psychiatric patients than among the Dutch population.
Schneider et al. (2000) [54]	Cross-sectional	DSM-III	Cases: 34	Female:15 Male: 19	38.9 ± 2.1	N/A	35.16 ± 26.1	Patients with schizophrenia had significantly lower 25(OH)D levels than healthy controls (*p* < 0.02). There was no significant difference between groups (schizophrenia, depression, alcoholism). The levels of 25(OH)D negatively correlated with the parathyroid hormone (*p* < 0.01).
Jamilian et al. (2013) [33]	Cross-sectional	DSM-IV	Cases: 100 Controls: 100	Female: 32 Male: 68	35.67 ± 10.46	N/A	47.00 ± 11.39	The serum vitamin D levels in healthy participants were significantly higher than in both depressed (*p* < 0.001) and schizophrenic (*p* = 0.001) patients. There was no significant difference between the vitamin D levels in the depressed and schizophrenic group (*p* = 0.563).
Gaebler et al. (2022) [55]	Cross-sectional	DSM-V	Cases: 141	Female: 40 Male: 101	33.1 ± 11.4	Yes	14.5 ± 8.9	A total of 78% of patients exhibited low vitamin D levels, with more pronounced cognitive impairments.
Zhu et al. (2015) [34]	Cross-sectional	DSM-IV	Cases: 93 Controls: 93	Female: 49 Male: 44	29.49 ± 9.93	No	10.55 ± 5.32	The mean 25(OH) levels were 39.6% lower for patients with schizophrenia compared to controls. Participants with a high CRP and low 25(OH)D had the highest prevalence of schizophrenia, while participants with a low CRP and high 25(OH)D had the lowest.
Ghosh et al. (2023) [35]	Cross-sectional	ICD-10	Cases: 50 Controls: 50	N/A	N/A (20–39 yo)	no	Median: 12.45 ng/mL	The median level of vitamin D was statistically significant (*p* = 0.009) among the cases (12.45 ng/mL) and among the controls (20.03 ng/mL).
Yazici et al. (2019) [36]	Cross-sectional	DSM-IV	Inpatient: 30 Outpatient: 30 Controls: 28	Male: 60	Inpatients: 40.63 ± 13.5 Outpatients: 40.50 ± 10.59	Yes	Inpatients: 16.20 ± 17.93 Outpatients: 11.37 ± 4.58	The study found no statistically significant differences in vitamin D levels between the control group and schizophrenia patients, both in acute and remission stages. The correlation between vitamin D level and PANSS score was not significant.
Yazici et al. (2019) [37]	Cross-sectional	DSM-V	Cases: 189 Controls: 109	Female: 81 Male: 108	41.44 ± 12.28	N/A	17.52 ± 9.65	The study found that 66.46% of schizophrenia subjects had vitamin D insufficiency and 21.95% had a deficiency, leading to an overall insufficiency/deficiency incidence of 88.41%. The vitamin D deficiency was significantly higher in the schizophrenia group than in the substance use disorder (*p* ≤ 0.001) and healthy control (*p* ≤ 0.05) groups.
Shahini et al. (2022) [38]	Case–control	DSM-V	Cases: 33 Controls: 33	Female: 8 Male: 25	Median 40 (34–47 yo)	N/A	Median (µg/dl) 5.3 (1.75–9.65)	Serum vitamin D levels were significantly lower in schizophrenic patients than in the general population (*p* = 0.035). The correlation between vitamin D level and PANSS score was not significant.
Yuan et al. (2020) [39]	Case–control	DSM-IV	Cases: 163 Controls: 75	Male/Female: Aripiprazole 24/27 Olanzapine 25/23 Paliperidone 17/16 Amisulpride 14/11 Nonmedicated 3/3	Aripiprazole 28.8 ± 11.1 Olanzapine 28.7 ± 7.9 Paliperidone 29.1 ± 9.3 Amisulpride 30.8 ± 9.8 Nonmedicated 29.7 ± 10.4	yes	Aripiprazole 13.5 ± 5.5 Olanzapine 10.4 ± 5.3 Paliperidone 10.7 ± 4.8 Amisulpride 7.7 ± 4.6 Non-medicated 8.1 ± 2.6	Significantly lower vitamin D concentrations were found in the non-medicated group compared with healthy controls after covariance analysis. Additionally, aripiprazole could affect vitamin D concentrations in vivo, and a positive correlation between aripiprazole concentrations and vitamin D concentrations (r = 0.319, *p* = 0.025) was seen in the aripiprazole group.
Bulut et al. (2016) [40]	Cross-sectional	DSM-IV	Cases: 80 Controls: 74	Female: 38 Male: 42	36.59 ± 9.96	N/A	23.46 ± 13.98	There was no significant difference in 25(OH)D levels between schizophrenia and healthy control groups. Lower vitamin D correlated with the occurrence of positive and negative symptoms along with an increased severity of the symptoms.
Humble et al. (2010) [56]	Cross-sectional	ICD-10	Cases: 20	Female: 12 Male: 8	47.4	N/A	Median 35 (23.5; 52.5)	Only 14.5% of the patients had recommended levels of 25-OHD, whereas 56.4% of patients had vitamin D levels under 50 nmol/l. There was a negative correlation between 25OHD and iPTH (*p* = 0.002).
Menkes et al. (2012) [57]	Cross-sectional	DSM-IV	Cases: 38	N/A	N/A	yes	13.8 ± 6.16	Vitamin D varied by diagnosis, with schizophrenia associated with markedly lower levels than mania and depression (*p* < 0.001). A total of 34% of schizophrenia patients had vitamin D deficiency, compared with 9.4% of other participants (*p* = 0.003).
Arya et al. (2023) [58]	Cross-sectional	DSM-IV	Cases: 49	Female: 10 Male: 39	24.92 ± 2.85	no	Median 10.8 (3.4–36.7)	There was no significant difference in vitamin D levels between schizophrenia and controls.
Partti et al. (2010) [59]	Cross-sectional	DSM-IV	Cases: 48	Female: 28 Male: 20	53.5 (95% Cl 52.2–56.7)	yes	15.59 (95% Cl 13.76–17.36)	Significantly lower vitamin D levels were observed in subjects with schizophrenia in comparison with the general population (*p* = 0.006). Women with schizophrenia had significantly lower bone ultrasound values compared with matched controls (*p* = 0.001).
Rajith et al. 2022 [41]	Cross-sectional	DSM-V	Cases: 74 Controls: 72	Female: 26 Male: 48	42.7 ± 13	yes	16.25 ± 5.5	There was no significant difference in the mean vitamin D levels between groups. Antipsychotic treatment had no significant effect on vitamin D.
Eskelinen at al. (2020) [60]	Cross-sectional	N/A	Cases: 275	Female: 85 Male: 190	44.9 ± 12.6	yes	21.24 ± 7.84	A total of 47% of schizophrenia patients had vitamin D levels below the reference range.
Van der Leeuw et al. (2020) [42]	Cross-sectional	DSM-IV	Cases: 347 Controls: 282	Female: 79 Male: 268	30.3 ± 6.9	yes	18.8 ± 10.44	Vitamin D concentrations were significantly lower in patients (*p* = 0.005). Urbanicity at birth was negatively associated with vitamin D concentration in schizophrenia (*p* = 0.020). Higher vitamin D was associated with a lower PANSS score.
Salavert et al. (2017) [43]	Cross-sectional; Case–control	DSM-IV	Cases: 22 Controls: 22 Other psychoses: 22	Female: 6 Male: 16	31.1 ± 9.2	no	13.14 ± 5.96	Schizophrenia had significantly lower levels of vitamin D compared with controls (*p* < 0.001). No significant differences were found between schizophrenia versus other psychoses groups.
Yoo et al. (2018) [61]	Cross-sectional	ICD-10	Cases: 302	Female: 134 Male: 168	40.7 ± 12.0	yes	15.5 ± 6.4	A total of 78.1% of patients had vitamin D insufficiency. Vitamin D insufficiency was significantly associated with metabolic syndrome (*p* = 0.006) and hypertension (*p* = 0.017).
Cieslak et al. (2024) [62]	Cross-sectional	N/A	Cases: 22	Female: 9 Male: 13	Female: 41.9: ± 9.6 Male: 44.3 ± 7.2	yes	17.3 ± 8.87	A total of 91% of schizophrenia patients had deficient or insufficient Vitamin D levels.
Kulaksizoglu et al. (2017) [44]	Cross-sectional	DSM-IV	Cases: 64 Controls: 54	Female: 28 Male: 36	38.25 ± 7.69	yes	10.06 ± 2.64	The schizophrenia patient group expressed lower vitamin D and brain-derived neurotrophic factor (BDNF) levels, and had a significant, positive correlation between BDNF and vitamin D levels (*p* = 0.017). Lower levels of vitamin D were found in the schizophrenia patient group with MetS compared with the patient group without metabolic syndrome (MetS); however, the difference was not significant.
Gaebler et al. (2022) [63]	Cross-sectional	DSM-V	Cases: 80	Female: 19 Male: 61	Median 30	yes	Median 12.6	A negative relationship has been observed between vitamin D and dose-adjusted antipsychotic drug concentrations, which was particularly pronounced for drugs which were predominantly metabolized via CYP3A4 (aripiprazole and quetiapine).
Lally et al. (2016) [64]	Cross-sectional	ICD-10	Cases: 218	N/A	N/A	N/A	11.5 ± 6.7	Individuals with non-affective psychosis had lower vitamin D levels than those with affective psychosis; however, the results were not significant (*p* = 0.061).
Itzhaky et al. (2012) [45]	Cross-sectional	N/A	Cases: 50 Controls: 33 Depression group: 50	Female: 16 Male: 34	40.2 ± 13.4	yes	15.0 ± 7.3	Lower serum vitamin D concentrations were detected among patients with schizophrenia (15.0 ± 7.3 ng/mL) compared with patients with depression (19.6 ± 8.3 ng/mL, *p* < 0.05) and controls (20.2 ± 7.8 ng/mL, *p* < 0.05). The correlation between vitamin D levels and the PANSS score was not significant.
Azar et al. (2017) [46]	Case–control	N/A	Cases: 100 Controls: 100	Female: 30 Male: 70	37.00 ± 11.65	yes	N/A (groups were divided into <25 ng/mL or >25 ng/mL 25(OH)D)	Schizophrenia patients had lower vitamin D levels compared with control group; the difference was not significant (*p* = 0.053).
Yüksel et al. (2014) [47]	Case–control	DSM-IV	Cases: remission: 41 acute: 40 Controls: 40	Remission Female: 14 Male: 27 Acute Female:20 Male:20	Remission: 38.85 ± 10.64 Acute: 38.08 ± 11.26		Median (25–75%) Remission: 15.03 (9.8–20.1) Acute: 15.02 (8–21.7)	Patients in an acute episode had significantly lower vitamin D levels compared with patients in remission and healthy controls (*p* < 0.0001). There were no significant differences between groups in terms of serum *p*, Ca and PTH levels. No significant impact of weekly duration of sun exposure on total vitamin D levels.
Clelland et al. (2014) [48]	Case–control	DSM-IV	Cases: 64 Controls: 90	Female: 33 Male: 31	38.5 ± 11.3	yes	31.63 ± 22.64	Patients with schizophrenia had significantly lower levels of 25(OH)D compared to matched controls (OR 2.1, adjusted *p* = 0.044, 95% CI: 1.02–4.46). The results suggest that over one third of the association between 25(OH)D insufficiency and schizophrenia may be explained by the presence of hyperprolinemia.
Ling et al. (2023) [65]	Cross-sectional	DSM-V	Cases: 118	Female:46 Male: 72	43.13 ± 10.17	yes	n/o (groups were divided into <30 ng/mL or >30 ng/mL 25(OH)D)	Patients with vitamin D insufficiency had higher bone resorption marker levels and lower bone formation marker levels compared with those with sufficiency. There was no direct association between vitamin D levels and overall cognitive function (RBANS).
Goh et al. (2024) [49]	Case–control	DSM-V	Cases: 150 Controls: 139	Female:70 Male: 80	42.6 ± 17.6	yes	n/o (groups were divided into <30 ng/mL (deficiency) and <20 ng/mL (insufficiency) 25(OH)D	Patients with schizophrenia had significantly lower serum vitamin D levels compared to healthy controls (*p* < 0.01), especially those taking atypical antipsychotics (*p* = 0.02) and those who were obese (BMI ≥ 27.5 kg/m^2^) (*p* = 0.04).
Akindale et al. (2017) [50]	Case–control	DSM-IV ICD-10	Cases: 60 Controls: 30	n/a	35.10 ± 9.15	yes	19.75 ± 5.19	Vitamin D was significantly lower in patients with schizophrenia compared with the controls (*p* < 0.05). There was no significant correlation between vitamin D level and PANSS scores (*p* = 0.66).

**Table 7:** Scores of symptom severity scales in relation to vitamin D status.

Study	Symptoms Scale	Effect	Notes
Shivakumar et al. (2015) [51]	SANS, SAPS	0	No significant relationship with vitamin D level.
Shahini et al. (2022) [38]	PANSS	0	No significant relationship with vitamin D level.
Itzhaky et al. (2012) [45]	PANSS	0	No significant relationship with vitamin D level.
Yazici et al. (2019) [37]	PANSS, CGI, GAF	0	No significant relationship with vitamin D level.
Akinlade et al. (2017) [50]	PANSS	0	No significant relationship with vitamin D level.
Prasanty et al. (2018) [52]	PANSS	-	Negative correlation between serum levels of vitamin D and the PANSS score in schizophrenic patients for positive PANSS score (*p* < 0.001), negative PANSS score (*p* < 0.001), general psychopathology (*p* < 0.001), and total PANSS score (*p* < 0.001).
Bulut et al. (2016) [40]	SANS, SAPS	-	Negative correlations found for SANS total points (*p* = 0.038), attention points (*p* = 0.044), and positive formal thoughts (*p* = 0.021)
Van der Leeuw et al. (2020) [42]	PANSS	-	Higher vitamin D concentration was associated with lower positive (*p* = 0.049) and negative symptom levels (*p* = 0.008).
Gaebler et al. (2022) [55]	TMT, BCRS	+	Patients with lower vitamin D levels exhibited more pronounced cognitive impairments, after regression analysis the impact of vitamin D remained only significant for TMT-A (*p*-corrected = 0.045).
Ling et al. (2023) [65]	RBANS	0	No significant relationship with vitamin D level.

**Table 8:** Effect of medication on vitamin D level.

Study	Medication	Effect Direction	Notes
Rajith et al. (2022) [41]	haloperidol, trifluoperazine, risperidone, olanzapine, clozapine	0	No significant influence on vitamin D level.
Itzhaky et al. (2012) [45]	non-specified antipsychotics	0	No significant influence on vitamin D level.
Yuan et al. (2020) [39]	non-medicated	–	Vitamin D level was significantly lower than in health controls (Mean 8.1 ± 2.6 ng/mL vs. 13.1 ± 5.2; *p* < 0.05). Small sample, *n* = 6.
	olanzapine, paliperidone, amisulpride	0	No significant influence on vitamin D level.
	aripiprazole	+	Positive correlation between aripiprazole plasma level and vitamin D (r = 0.319, *p* = 0.025).
Goh et al. (2024) [49]	atypical antipsychotics, combined antipsychotics	–	Atypical (*p* = 0.02) and combined (*p* = 0.02) antipsychotic users had significantly lower vitamin D levels compared to control.
Gaebler et al. (2022) [63]	aripiprazole, quetiapine	–	Negative association between vitamin D concentration and antipsychotics predominantly metabolized by CYP3A4. The drug metabolism with aripiprazole (*p* = 0.031) and quetiapine (*p* = 0.006) exhibited the highest anti-correlation. Patients above median vitamin D more often had aripiprazole/quetiapine levels below therapeutic range.
	clozapine, risperidone, amisulpride, olanzapine	-	Non-significant inverse trend: amisulpride (*p* = 0.298), olanzapine (*p* = 0.228), clozapine (*p* = 0.58), and risperidone (*p* = 0.345).

## Abbreviations

The following abbreviations are used in this manuscript:

1,25-(OH)2D	calcitriol
DBP	Vitamin D-binding protein
25(OH)D	Calcidiol
VDR	Vitamin D receptor
IU	International unit
SPF	Sun protection factor
PANSS	Positive and negative syndrome scale
SANS	Scale for the assessment of negative symptoms
SAPS	Scale for the assessment of positive symptoms
BACS	Brief assessment of cognition in schizophrenia
TMT	Trail making test
DSM	Diagnostic and statistical manual of mental disorders
ICD	International classification of diseases
EPS	Extrapyramidal symptoms
PTH	Parathyroid hormone
MDD	Major depressive disorder
CRP	C-reactive protein
BDNF	Brain-derived neurotrophic factor
MetS	Metabolic syndrome
